# Exploration on OCT biomarker candidate related to macular edema caused by diabetic retinopathy and retinal vein occlusion in SD-OCT images

**DOI:** 10.1038/s41598-024-63144-2

**Published:** 2024-06-21

**Authors:** Yuhui Tao, Lexin Ge, Na Su, Mingchao Li, Wen Fan, Lin Jiang, Songtao Yuan, Qiang Chen

**Affiliations:** 1 School of Computer Science and Engineering, Nanjing University of Science and Technology, No.200 Xiao Lingwei, Nanjing, 210094 China; 2 Department of Ophthalmology, The First Affiliated Hospital of Nanjing Medical University, No.300 Guangzhou Road, Nanjing, 210029 China; 3 Department of Endocrinology, The First Affiliated Hospital of Nanjing Medical University, Nanjing, China

**Keywords:** Biomarkers, Diseases

## Abstract

To improve the understanding of potential pathological mechanisms of macular edema (ME), we try to discover biomarker candidates related to ME caused by diabetic retinopathy (DR) and retinal vein occlusion (RVO) in spectral-domain optical coherence tomography images by means of deep learning (DL). 32 eyes of 26 subjects with non-proliferative DR (NPDR), 77 eyes of 61 subjects with proliferative DR (PDR), 120 eyes of 116 subjects with branch RVO (BRVO), and 17 eyes of 15 subjects with central RVO (CRVO) were collected. A DL model was implemented to guide biomarker candidate discovery. The disorganization of the retinal outer layers (DROL), i.e., the gray value of the retinal tissues between the external limiting membrane (ELM) and retinal pigment epithelium (RPE), the disrupted and obscured rate of the ELM, ellipsoid zone (EZ), and RPE, was measured. In addition, the occurrence, number, volume, and projected area of hyperreflective foci (HRF) were recorded. ELM, EZ, and RPE are more likely to be obscured in RVO group and HRFs are observed more frequently in DR group (all *P* ≤ 0.001). In conclusion, the features of DROL and HRF can be possible biomarkers related to ME caused by DR and RVO in OCT modality.

## Introduction

Macular edema (ME) is the accumulation of fluid in the retina of the macular area, which can cause moderate to severe visual impairment^1^. ME can occur in a variety of clinical diseases, such as diabetic retinopathy (DR), retinal vein occlusion (RVO), age-related macular degeneration, and uveitis. In this study, we mainly focused on DR and RVO, which are two major causes of vision loss in working age people in developed countries^2,3^.

Spectral-domain optical coherence tomography (SD-OCT) imaging technology can obtain high resolution 3D retinal images by acquiring a series of cross-sectional slices (B-scans) in a fast and noninvasive way. It has played an important role in the quantitative analysis of retinal fluid and gained increasing popularity as an objective tool to measure retinal thickness and other aspects associated with ME.

Over the past few years, several effective treatments for ME, such as anti-vascular endothelial growth factor (anti-VEGF) therapy^4^ and dexamethasone intravitreal implants^5^, have been developed. However, some ME cases show suboptimal response to these treatments and the relationship between characteristics of ME and its treatment response remains unclear, as the cause of ME is complex and incompletely understood. At present, most studies focused on discovering postoperative factors related to visual acuity (VA) in subjects diagnosed with DR or/and RVO^6–10^ (shown in Table 1). Indicators related to VA include disorganization of the retinal inner layers (DRIL), hyperreflective foci (HRF), the integrity of external limiting membrane (ELM) and ellipsoid zone (EZ), etc. However, to the best of our knowledge, few studies attempted to find biomarkers to distinguish between DR and RVO, which means that the cause of ME has not been fully explored. Balaratnasingam et al.^10^ mentioned the difference between DR and RVO, such as DRIL and EZ disruption; but they did not discuss this issue in depth. If we can find the biomarker related to the pathogenesis of the ME and further observe whether it can reflect the efficacy of injection, this may help elucidate the pathophysiology of the disease, which will in turn help predict the treatment response and reduce the unnecessary treatment.

**Table 1:** Comparison with Related Studies Based on OCT Images.

Related works	Eyes	Purpose	Main findings
Sun^6^	120 DR	To find indicators related to VA	DRIL is correlated with VA
Zur^7^	299 DR	To find indicators related to VA	HRF and the integrity of EZ are correlated with VA
Tang^8^	63 RVO	To find indicators related to VA	The integrity of ELM and EZ is correlated with VA
Yiu^9^	202 RVO	To find indicators related to VA	DRIL, CST, and the integrity of ELM and EZ are correlated with VA
Balaratnasingam^10^	65 DR, 30 RVO	To find indicators related to VA	FAZ area is correlated with VA. VA (*P* = 0.974), PFT (*P* = 0.050), CST (*P* = 0.056), DRIL (*P* = 0.252), and EZ disruption (*P* = 0.473) are not significantly different between DR and RVO groups. FAZ area is greater in DR group (*P* = 0.019). Intraretinal cysts occur in RVO group more frequently (*P* = 0.033)
Ours	109 DR, 137 RVO	To find biomarkers related to DR and RVO	ELM disruption (*P* = 0.323) and EZ disruption (*P* = 0.823) are not significantly different between DR and RVO groups. ELM, EZ, and RPE obscuring, and HRF features are significantly different between DR and RVO groups (all *P* < 0.001)

*DR* diabetic retinopathy, *RVO* retinal vein occlusion, *VA* visual acuity, *DRIL* disorganization of the retinal inner layers, *HRF* hyperreflective foci, *CST* central 1-mm subfield thickness, *FAZ* foveal avascular zone, *PFT* point thickness of the central fovea, *ELM* external limiting membrane, *EZ* ellipsoid zone, *RPE* retinal pigment epithelium.

*P* values were considered significant at ≤ 0.05.

With the rise of deep learning (DL), it has provided a powerful tool to identify, localize and quantify pathological features in retinal diseases^11^. In this paper, we investigated whether the DL model learned from big data can guide biomarker discovery and advance our clinical insight of the underlying pathophysiology beyond conventional knowledge. We find that the DL model mostly highlights the outer retina regions in OCT volumes, which hints that there may exist differences in characteristics of ME in outer retina layers between these two diseases. Based on this observation, we further measured the features of disorganization of the retinal outer layers (DROL). Besides, we analyzed the occurrence, number, volume, and projected area of HRF. We show that there is a strong significant difference in the characteristics of DROL and HRF between DR and RVO groups.

## Methods

### Patient characteristics

In this study, 218 patients diagnosed with diabetic macular edema (DME) and macular edema secondary to retinal vein occlusion (RVO-ME) who underwent fluorescein fundus angiography (FFA) examination with SD-OCT images between November 2020 and August 2022 at Jiangsu Province Hospital were retrospectively analyzed. The dataset was collected with Cirrus SD-OCT device (Zeiss Meditec). The size of a full SD-OCT scan is 1024 × 512 × 128 and corresponds to the trim size of 2 mm × 6 mm × 6 mm in the axial (z), horizontal (x) and vertical (y) directions, respectively. The overview of the dataset is provided in Supplementary Table S1. A total of 246 eyes of 218 subjects from a single visit were analyzed. The cohort comprised 109 eyes with DR (32 with non-proliferative diabetic retinopathy (NPDR) and 77 with proliferative diabetic retinopathy (PDR)) and 137 eyes with RVO (120 with branch retinal vein occlusion (BRVO) and 17 with central retinal vein occlusion (CRVO)). This study was approved by the ethic committee of the First Affiliated Hospital with Nanjing Medical University (2017-SR-283) and conducted in accordance with the tenets of the Declaration of Helsinki. As this is a retrospective study with data processed anonymously, informed consent was waived by the ethic committee of the First Affiliated Hospital with Nanjing Medical University. The ethic committee approved the experiments, including any relevant details and confirmed that all experiments were performed in accordance with relevant guidelines and regulations.

### Inclusion criteria

Patients met the following requirements were included in this study: (1) treatment-naive patients diagnosed as ME secondary to RVO or DR based on the results of FFA and OCT examination; (2) older than 18 years; (3) with intraocular pressure < 21mmhg (millimeters of mercury) and binocular difference < 5mmhg, and without medical history of ocular hypertension.

### Exclusion criteria

Patients with the following characteristics were excluded in this study: (1) with type 1 diabetes; (2) with optic disc vasculitis; (3) with unclear SD-OCT images due to the opacification of refractive stroma, such as corneal lesions, cataract, vitreous hemorrhage, etc.; (4) with severe carotid artery occlusion, stenosis or other diseases caused by ophthalmic artery occlusion, such as retinal artery occlusion and ocular ischemic syndrome; (5) with systemic diseases that affect OCT imaging, such as autoimmune diseases, parkinsonism, hypertension, liver and kidney dysfunction, etc.

### Deep learning model architecture and training

The primary purpose of this study is to discover biomarkers related to DR and RVO; therefore, the NPDR and PDR groups and BRVO and CRVO groups were first pooled together to a single DR group and a RVO group for model training to guide biomarkers discovery. We employed a convolutional neural network to automatically distinguish between DR and RVO SD-OCT volumes, and then resorted to feature visualization techniques to highlight the most discriminative parts of OCT images to guide biomarker discovery. More specifically, we used a variant of 3D ResNet^12^ named R(2 + 1)D^13^. We utilized R(2 + 1)D-18 which consists of 18 convolution layers, one fully connected layer and one global average pooling layer between convolutional layers and fully connected layer.

During training process, the spatial resolution of SD-OCT images was resized into the size of 266 × 138 due to the hardware limitation and we did not down-sample along the vertical direction. We further randomly cropped them to 256 × 128, horizontally flipped and rotated them from (-15°, 15°) in horizontal and axial directions for data argumentation. The model was trained with standard cross entropy loss for 100 epochs, and we utilized Adam optimizer with a batch size of 4 and a learning rate of 5e-4. In test phase, we directly rescaled the test input to 256 × 128.

We performed fivefold cross-validation experiment for performance evaluation. Note that there were no overlapping patients in the training set and testing set. Besides, to reduce the inductive bias of DL model, we manually partitioned the dataset to ensure the data diversity in each fold.

### Saliency maps generation

Once the training procedure is finished, we evaluated the trained model on test set in each fold, and the DL model is supposed to explain its decision base in addition to its prediction. Here, we utilized Grad-CAM (Gradient-weighted Class Activation Mapping)^14^ which can highlight the discriminative areas of different categories given the classification label. The original attention maps were grayscale images, where the pixel values indicate the contribution of each pixel to the classification outcome. We performed min–max normalization on the attention map volume and scaled it to a range from 0 to 1. Then, for each 2D slice in an attention map volume, we upsampled it to 1024 × 512 and overlaid it on the corresponding B-scan in the form of heatmap to obtain pseudo-color image as final saliency map. We recorded the highlighted regions and manually proposed a novel clinical parameter inspired by the statistical results of attention map distribution, named disorganization of the retinal outer layers (DROL), which will be discussed later.

### OCT-derived measurements

The mean and standard deviation (std) were calculated for each DROL and HRF features, separately for DR and RVO groups as well as their subgroups. The distributions of the above features were non-normal, as examined using the Shapiro–Wilk test; therefore, nonparametric test, i.e., the Mann–Whitney U test was used to compare the observed features between DR and RVO groups. Statistical significance was set at *P* ≤ 0.05 and the alpha level for all tests was set as 0.05. All analysis was performed using Matlab R2021b software (The MathWorks, Inc., Natick, MA). The OCT-derived measurements are described as follows.

1. Saliency regions obtained by DL model: We combined the observations from 3D rendered images (as our purpose is to find which part of OCT volume is disease-specific) with the results from 2D slices (as retinal layer structures are more clearly visible in 2D results) as the final evaluation of OCT volumes for statistical analysis. As shown in Fig. 1a, the total retina (TR) is divided into inner retina (IR; the retinal region between inner limiting membrane (ILM) and outer plexiform layer (OPL)) and outer retina (OR; the retinal region between OPL and Brunch's membrane (BM)). The distribution of saliency regions was analyzed based on this kind of division for the following two reasons:

**Figure 1:**
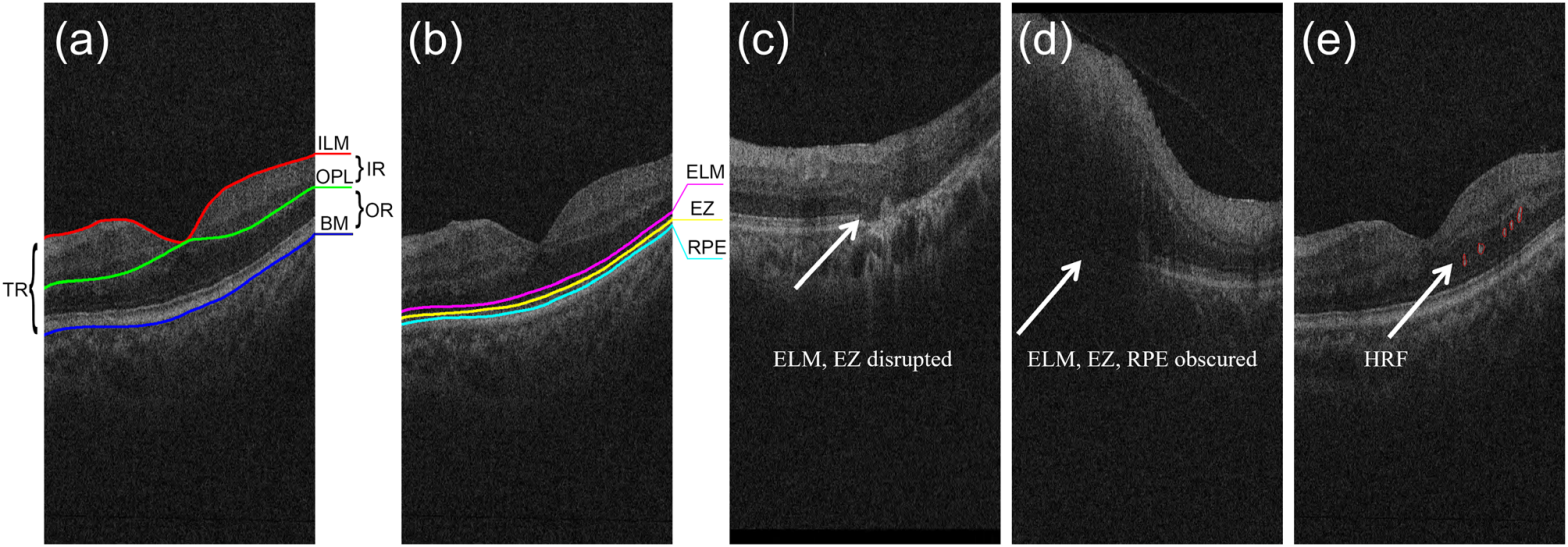
Illustration of layer segmentations, DROL, and HRF. (**a**) An example with ILM, OPL, and BM segmentation. TR is divided into IR and OR based on these layer segmentations. (**b**) An example with integrated ELM, EZ, and RPE. (**c**) An example with ELM and EZ disrupted. (**d**) an example with ELM, EZ, and RPE obscured. (**e**) An example with HRFs (in red circles). DROL = disorganization of the retinal outer layers; HRF = hyperreflective foci; ILM = inner limiting membrane; OPL = outer plexiform layer; BM = Brunch's membrane; TR = total retina; IR = inner retina; OR = outer retina; ELM = external limiting membrane; EZ = ellipsoid zone; RPE = retinal pigment epithelium.
  Previous clinical research^7–9^ has demonstrated a significant correlation between the integrity of the OR layers (especially ELM and EZ) and visual prognosis.
  Although obtained attention maps can provide a rough localization of regions related to disease categories, their positioning is often imprecise. Therefore, conducting a more detailed analysis of attention map distribution in a specific retinal region (for example, region between ELM and EZ) introduces potential errors beyond human factors.
  Besides, to mitigate the negative impacts of subjective interpretation on saliency regions, two observers (Y. Tao and M. Li) recorded which region was highlighted by the DL model independently. In cases where there were discrepancies in the results, a third researcher (Q. Chen) was consulted to make a final determination.
2. Features of disorganization of the retinal outer layers (DROL):
  i Average gray value of retinal regions between ELM and retinal pigment epithelium (RPE): Layer segmentation results of ELM and RPE were first obtained by a DL based model^15^ and then corrected by two independent examiners (L. Ge and N. Su) and the average measurement was used as gold standard.
  ii Integrity of ELM, EZ and RPE (Fig. 1b): Each B-scan image in an SD-OCT volume was categorically graded as being integrated, disrupted, or obscured by two independent graders (L. Ge and N. Su). When the evaluation was inconsistent between the two graders, a third masked reader (S. Yuan) made the final arbitration. The disrupted rate of a specific layer was defined as the ratio of number of B-scans graded as being disrupted to that of all B-scans in one SD-OCT volume, similarly for the obscured rate. If a layer cannot be identified, and its surrounding structure is relatively clear, then this layer is disrupted (Fig. 1c); if its surrounding structure is completely invisible, then this layer is obscured (Fig. 1d).
3. Occurrence, number, volume, and projected area of hyperreflective foci (HRF): The bright spots (Fig. 1e) were first segmented by an automatic algorithm^16^ and then revised by two independent observers (L. Ge and N. Su). When the result was inconsistent between the two graders, a third masked specialist (S. Yuan) made the final arbitration. The occurrence of HRF was defined as the number of B-scans with HRF in one OCT volume. The projected area of HRF was defined as the area of HRF in the projection map.

## Results

### Classification performance of deep learning model

Balanced accuracy (BACC) and area under the curve (AUC) were utilized to measure the classification performance of the DL model. BACC is denoted as:

1 $$BACC=\frac{1}{k}\sum_{i=1}^{k}\sum_{j=1}^{{m}_{i}}1({\widehat{y}}_{ij}={y}_{ij})$$

where $k$ is the number of classes ($k$=2 in this paper), ${m}_{i}$ is the number of samples from the $i$-th class, $\widehat{y}$ is the ground truth and $y$ is the prediction of the DL model, $1(\cdot )\upepsilon \{0, 1\}$ is an indicator function that returns 1 if the input is true. The classification result is shown in Supplementary Table S2. The trained DL model can achieve a satisfactory result with an average 0.906 BACC and 0.937 AUC over 5-fold cross-validation.

### Analysis on saliency maps generated from deep learning model

The class saliency map was obtained based on the class label; therefore, only the OCT volumes predicted correctly (i.e., 94 DR and 130 RVO) were utilized with Grad-CAM to generate saliency maps for statistical analysis. The summary of highlighted retina regions is shown in Supplementary Fig. S1. Most samples were highlighted with outer retina (89% in DR group and 64% in RVO group).

**Figure 2:**
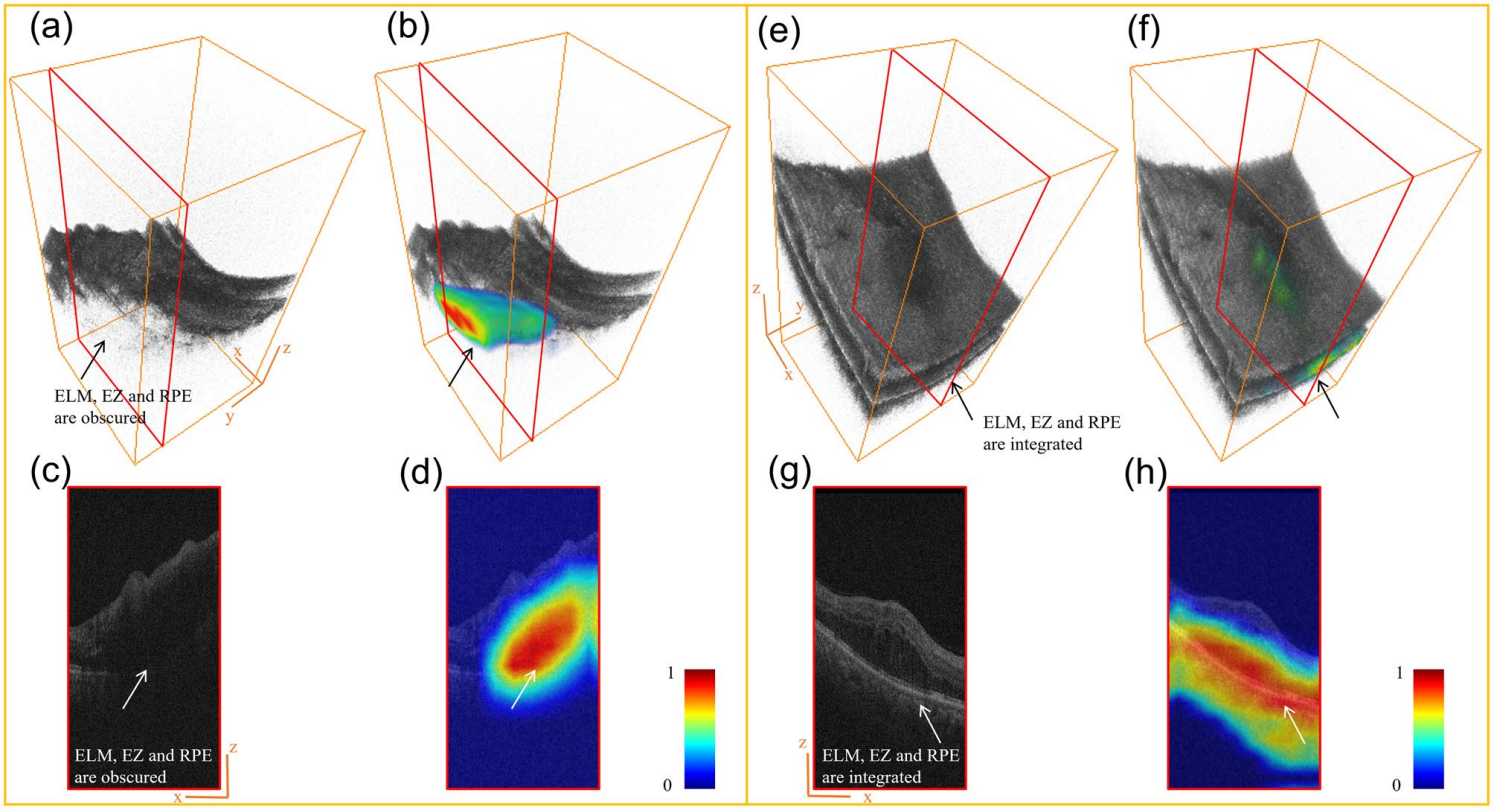
One RVO example (**a**–**d**) and one DR example (**e**–**h**) of OCT volume with heat map. The heat maps are generated by mapping the gray saliency maps into pseudo color images according to the gray level. (**a**) 3D rendering of a RVO volume. (**b**) 3D rendering of a RVO volume with heat map. (**c**) A 2D RVO B-scan. (**d**) A 2D RVO B-scan with heat map. (**e**) 3D rendering of a DR volume. (**f**) 3D rendering of a DR volume with heat map. (**g**) A 2D DR B-scan. (**h**) A 2D DR B-scan with heat map. DR = diabetic retinopathy; RVO = retinal vein occlusion; OCT = optical coherence tomography.

A qualitative comparison of saliency maps from a patient in RVO group (Fig. 2(a)-(d)) and another in DR group (Fig. 2e–h) is shown in Fig. 2. The 3D rendering of OCT volumes was presented using software Amira (FEI Company, Hillsboro, OR). The RVO volume was observed with obscured ELM, EZ, and RPE (Fig. 2a), which was highlighted by the DL model (Fig. 2b). On the other hand, the DR volume was observed with integrated ELM, EZ, and RPE (Fig. 2e), which was highlighted by the DL model (Fig. 2f) as well. The 2D B-scans (Fig. 2c and g) and 2D B-scans with heat maps (Fig. 2d and h) were also presented for a clearer illustration. In conclusion, the DL model mostly highlighted the outer retina regions to distinguish these two diseases, inspiring us to further analyze the characteristics of DROL.

### Comparison of DROL and HRF between DR and RVO groups

**Figure 3:**
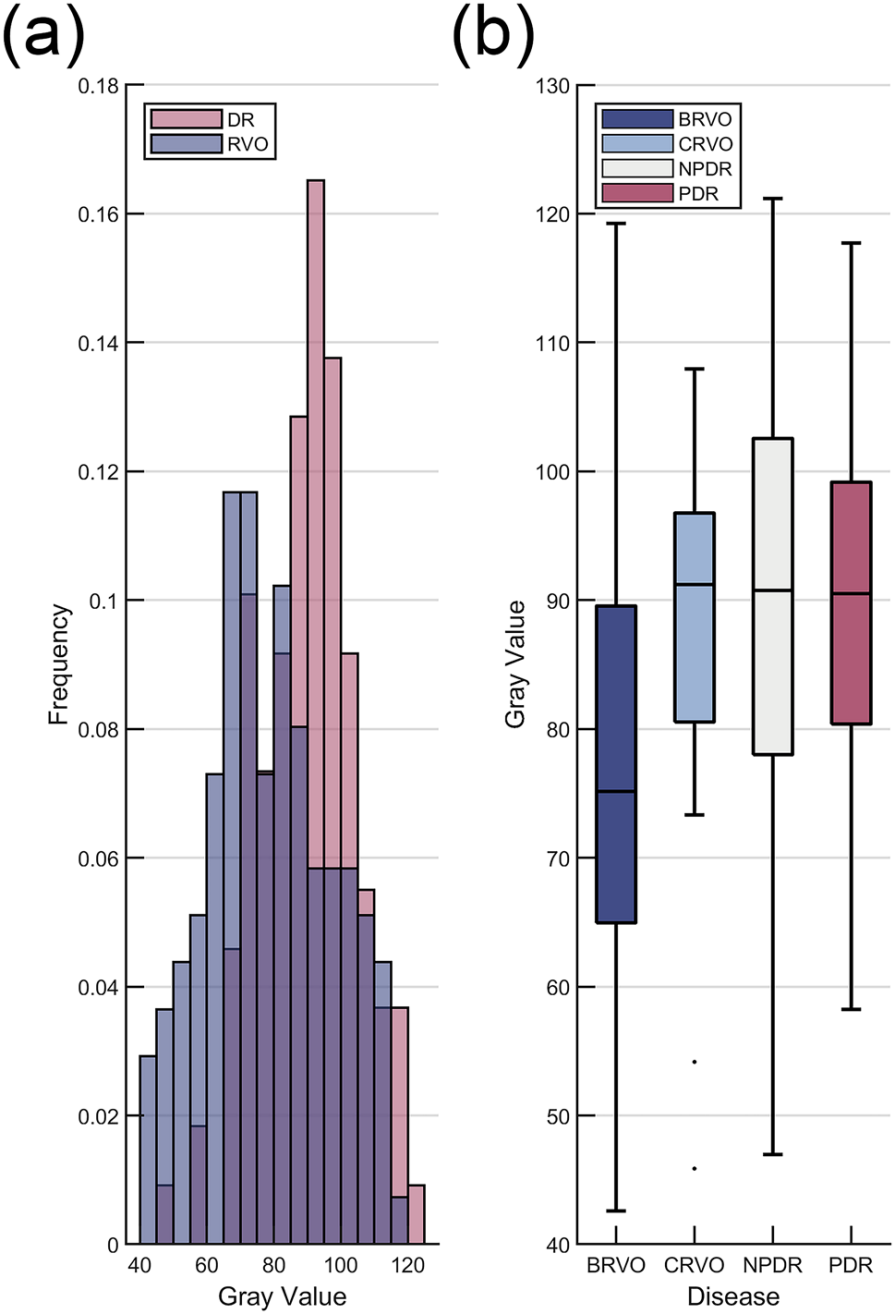
Distribution of average gray value of retinal regions from ELM to RPE of RVO and DR groups (**a**, histogram), and BRVO, CRVO, NPDR, and PDR subgroups (**b**, boxplots). The boxplots show the median and interquartile range. The range of whiskers is defined as [${q}_{3}$+1.5 × (${q}_{3}-{q}_{1}$), ${q}_{3}-$1.5 × (${q}_{3}{-q}_{1}$)], where ${q}_{3}$ and ${q}_{1}$ is 75th and 25th percentiles, respectively. The cases beyond this range are considered as outliers, which are individually shown. ELM = external limiting membrane; RPE = retinal pigment epithelium; RVO = retinal vein occlusion; DR = diabetic retinopathy; BRVO = branch RVO; CRVO = central RVO; NPDR = non-proliferative DR; PDR = proliferative DR.

We first investigated the difference between the gray value of retinal regions between ELM and RPE from DR and RVO groups. The histogram of distribution of gray value is shown in Fig. 3. The mean gray value of retinal regions from ELM to RPE in DR and RVO group is 90 (std, 15) and 79 (std, 19), respectively, and is significantly different (*P* < 0.001).

**Figure 4:**
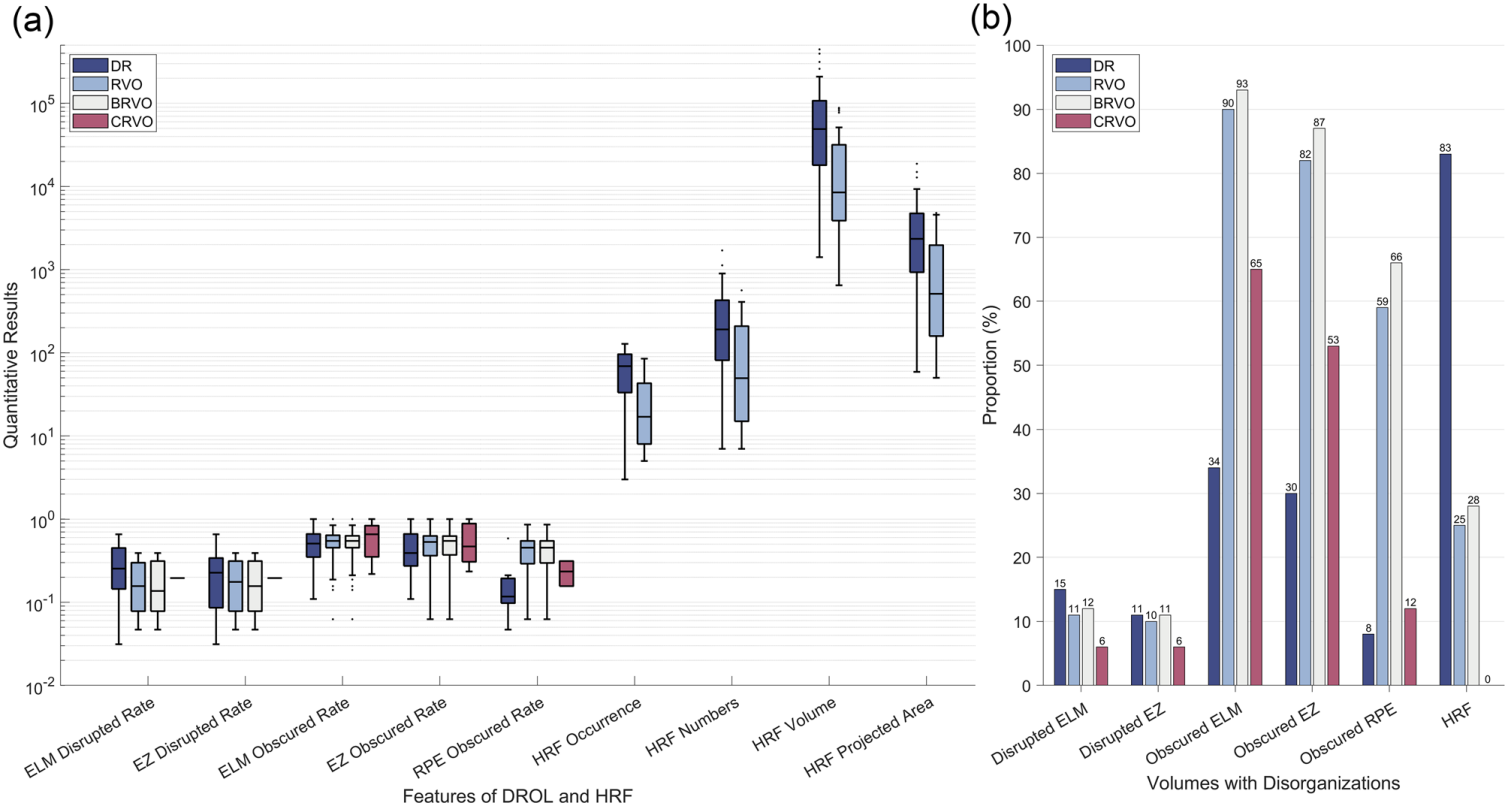
Distribution of features of DROL and HRF stratified by disease groups/subgroups DR, RVO, BRVO, and CRVO. The boxplots (**a**) show the median and interquartile range. The range of whiskers is defined as [${q}_{3}$+1.5 × (${q}_{3}-{q}_{1}$), ${q}_{3}-$1.5 × (${q}_{3}-{q}_{1}$)], where ${q}_{3}$ and ${q}_{1}$ is 75th and 25th percentiles, respectively. The cases beyond this range are considered as outliers, which are individually shown. The quantitative results are distributed across a logarithmic scale. Note that only volumes observed with a specific disorganization (i.e., not integrated layers or HRF) are presented in (**a**) for a clearer illustration. The boxes of CRVO subgroup are not presented in terms of HRF, as CRVO groups were not observed with any HRF, and only RVO group is presented (the boxes of BRVO are same as RVO). The bar graphs (**b**) show the proportion of volumes observed with a specific disorganization in each group/subgroup. DROL = disorganization of the retinal outer layers; HRF = hyperreflective foci; DR = diabetic retinopathy; RVO = retinal vein occlusion; BRVO = branch RVO; CRVO = central RVO; ELM = external limiting membrane; EZ = ellipsoid zone; RPE = retinal pigment epithelium.

We further analyzed the integrity of ELM, EZ, and RPE. The distributions of DROL and HRF features stratified by disease groups/subgroups are shown in Fig. 4. Note that only volumes observed with disorganizations are presented in Fig. 4a. From Fig. 4b, we observed that the proportion of OCT volumes with ELM (15% DR and 11% RVO volumes) or EZ disrupted is close (11% DR and 10% RVO volumes) between DR and RVO groups and is not significantly different (both *P* ≥ 0.323). On the other hand, the proportion of OCT volumes with ELM (34% DR and 90% RVO volumes), EZ (30% DR and 82% RVO volumes), or RPE obscured (8% DR and 59% RVO volumes) are all significantly different between these two groups (all *P* < 0.001). These three layers are more likely to be obscured in RVO group and lead to the lower gray value between ELM and RPE.

Besides the characteristics of DROL, we also roughly analyzed the pathological features of HRF. As shown in Fig. 4b, more volumes are observed with HRF in DR (83%) than in RVO group (25%). In the boxplots of number, volume, and projected area of HRF (Fig. 4a), the values of all four statistical results in DR group are larger than those in RVO group (all *P* < 0.001).

### Subgroup analysis on DROL and HRF

We further carried out subgroup analysis to see whether the above findings (i.e., there exists significant difference in the ELM, EZ, and RPE obscured rate and HRF features) are true under different conditions. We first analyzed the difference in inter-subgroups. As shown in Tables 2 and 3, on one hand, the value of all features between NPDR and PDR subgroups is close (all *P* ≥ 0.301); therefore, these two subgroups pooled into a single DR group for further comparison. On the other hand, the EZ and RPE obscured rate, average gray value from ELM to RPE as well as the HRF features show difference between BRVO and CRVO subgroups (all *P* < 0.05); therefore, these two subgroups were compared with DR group individually.

**Table 2:** Quantitative Results of DROL and HRF (mean ± std).

Features	Group/Subgroup					
	DR	PDR	NPDR	RVO	BRVO	CRVO
DROL						
ELM disrupted rate	0.04 ± 0.13 (15%)	0.03 ± 0.11 (14%)	0.06 ± 0.16 (16%)	0.02 ± 0.07 (11%)	0.02 ± 0.07 (12%)	0.01 ± 0.05 (6%)
EZ disrupted rate	0.03 ± 0.10 (11%)	0.02 ± 0.09 (12%)	0.03 ± 0.11 (10%)	0.02 ± 0.07 (10%)	0.02 ± 0.07 (11%)	0.01 ± 0.05 (6%)
ELM obscured rate	0.19 ± 0.31 (34%)	0.17 ± 0.29 (32%)	0.22 ± 0.35 (37%)	0.49 ± 0.25 (90%)	0.51 ± 0.23 (93%)	0.40 ± 0.38 (65%)
EZ obscured rate	0.15 ± 0.28 (30%)	0.14 ± 0.26 (30%)	0.19 ± 0.34 (31%)	0.42 ± 0.28 (82%)	0.44 ± 0.26 (87%)	0.30 ± 0.36 (53%)
RPE obscured rate	0.01 ± 0.07 (8%)	0.02 ± 0.08 (10%)	0.01 ± 0.02 (3%)	0.25 ± 0.25 (59%)	0.28 ± 0.25 (66%)	0.03 ± 0.08 (12%)
Average gray value	90 ± 15	90 ± 13	90 ± 18	79 ± 19	78 ± 19	86 ± 16
Characteristics of HRF						
Occurrence	55 ± 41 (83%)	55 ± 42 (84%)	54 ± 39 (81%)	7 ± 16 (25%)	8 ± 17 (28%)	0 ± 0 (0%)
Numbers	238 ± 274	230 ± 238	258 ± 351	31 ± 90	35 ± 95	0 ± 0
Volume	62,830 ± 80,514	58,702 ± 72,369	72,763 ± 97,982	5823 ± 17,551	6647 ± 18,615	0 ± 0
Projected area	2727 ± 3262	2590 ± 2828	3058 ± 4158	320 ± 925	365 ± 980	0 ± 0

*DR* diabetic retinopathy, *RVO* retinal vein occlusion, *NPDR* non-proliferative DR, *PDR* proliferative DR, *BRVO* branch RVO, *CRVO* central RVO, std standard deviation, *DROL* disorganization of the retinal outer layers, *HRF* hyperreflective foci, *ELM* external limiting membrane, *EZ* ellipsoid zone, *RPE* retinal pigment epithelium.

The percentage means the proportion of volumes observed with a specific disorganization in each group/subgroup.

**Table 3:** Comparison between Group/Subgroups.

Features	Manne-Whitney U Test P Value				
	DR & RVO	PDR & NPDR	BRVO & CRVO	DR & BRVO	DR & CRVO
DROL					
ELM disrupted rate	0.323	0.738	0.489	0.431	0.310
EZ disrupted rate	0.823	0.816	0.539	0.949	0.516
ELM obscured rate	< 0.001	0.628	0.213	< 0.001	< 0.05
EZ obscured rate	< 0.001	0.682	< 0.05	< 0.001	< 0.05
RPE obscured rate	< 0.001	0.301	< 0.001	< 0.001	0.589
Average gray value	< 0.001	0.806	< 0.05	< 0.001	0.587
Characteristics of HRF					
Occurrence	< 0.001	0.764	< 0.05	< 0.001	< 0.001
Numbers	< 0.001	0.897	< 0.05	< 0.001	< 0.001
Volume	< 0.001	0.924	< 0.05	< 0.001	< 0.001
Projected area	< 0.001	0.921	< 0.05	< 0.001	< 0.001

*DR* diabetic retinopathy, *RVO* retinal vein occlusion, *NPDR* non-proliferative DR, *PDR* proliferative DR, *BRVO* branch RVO, *CRVO* central RVO, *DROL* disorganization of the retinal outer layers, *HRF* hyperreflective foci, *ELM* external limiting membrane, *EZ* ellipsoid zone, *RPE* retinal pigment epithelium.

*P* values were considered significant at ≤ 0.05.

As shown in Fig. 4, the disrupted rate of ELM (12% BRVO and 6% CRVO volumes) and EZ (11% BRVO and 6% CRVO volumes) is similar in BRVO and CRVO subgroups; these two features remain insignificant in BRVO & DR and CRVO & DR comparisons (both* P* > 0.310). As shown in Fig. 3, the mean gray value of retinal regions from ELM to RPE in BRVO and CRVO group is 78 (std, 19) and 86 (std, 16), respectively, and the latter is close to the value in DR group (*P* = 0.587 in DR & CRVO comparison and *P* < 0.001 in DR & CRVO comparison). This is because RPE in CRVO volumes is less likely to be obscured (12%) compared with that in BRVO volumes (66%), and the difference between DR and CRVO cases is not significant (*P* = 0.589). However, a higher proportion of CRVO volumes are observed with obscured ELM (65% CRVO and 34% DR volumes) and EZ (53% CRVO and 30% DR volumes) compared with DR volumes (both *P* < 0.05).

From the aspect of HRF, none of 17 CRVO volumes were observed with HRF while 28% BRVO volumes were with HRF. In other words, RVO volumes are less likely to be observed with HRF compared with DR volumes (all *P* < 0.001).

## Discussion

We employed a DL model to automatically categorize ME caused by DR and RVO, and further utilized the knowledge learned from DL to guide biomarker candidate discovery. We did not target DL model as a substitute for ophthalmologists, but as a powerful tool for ophthalmologists to mine potential biomarkers^17^. To the best of our knowledge, this study is the first work tries to find biomarkers to distinguish ME caused by DR and RVO in OCT modality with the help of DL model. The main findings of this study are as follows: (1) The DL model can accurately classify DR and RVO SD-OCT volumes and guide biomarker discovery; (2) The integrity of ELM, EZ, and RPE is significantly different between DR and RVO groups; these three layers in RVO group are more likely to be obscured; (3) The characteristics of HRF are significantly different between DR and RVO groups; DR volumes have great chances to carry HRFs and the occurrence, number, volume, and projected area of HRF are greater in DR group.

The underlying mechanisms of ME are currently not well understood. Research on biomarkers in secondary macular edema associated with RVO and DR can help explore specific pathogenic mechanisms and provide relevant insights for subsequent treatment choices and improvement of visual outcomes. It is now believed that ME arises from two main factors^18–20^. Firstly, increased pressure after vascular occlusion leads to fluid leakage from blood vessels into adjacent retinal tissue. Secondly, endothelial cell damage in the affected veins may induce chronic inflammation in the retinal microvascular system, increase inflammatory mediators, and disrupt the blood-retinal barrier (BRB), ultimately resulting in ME.

**Figure 5:**
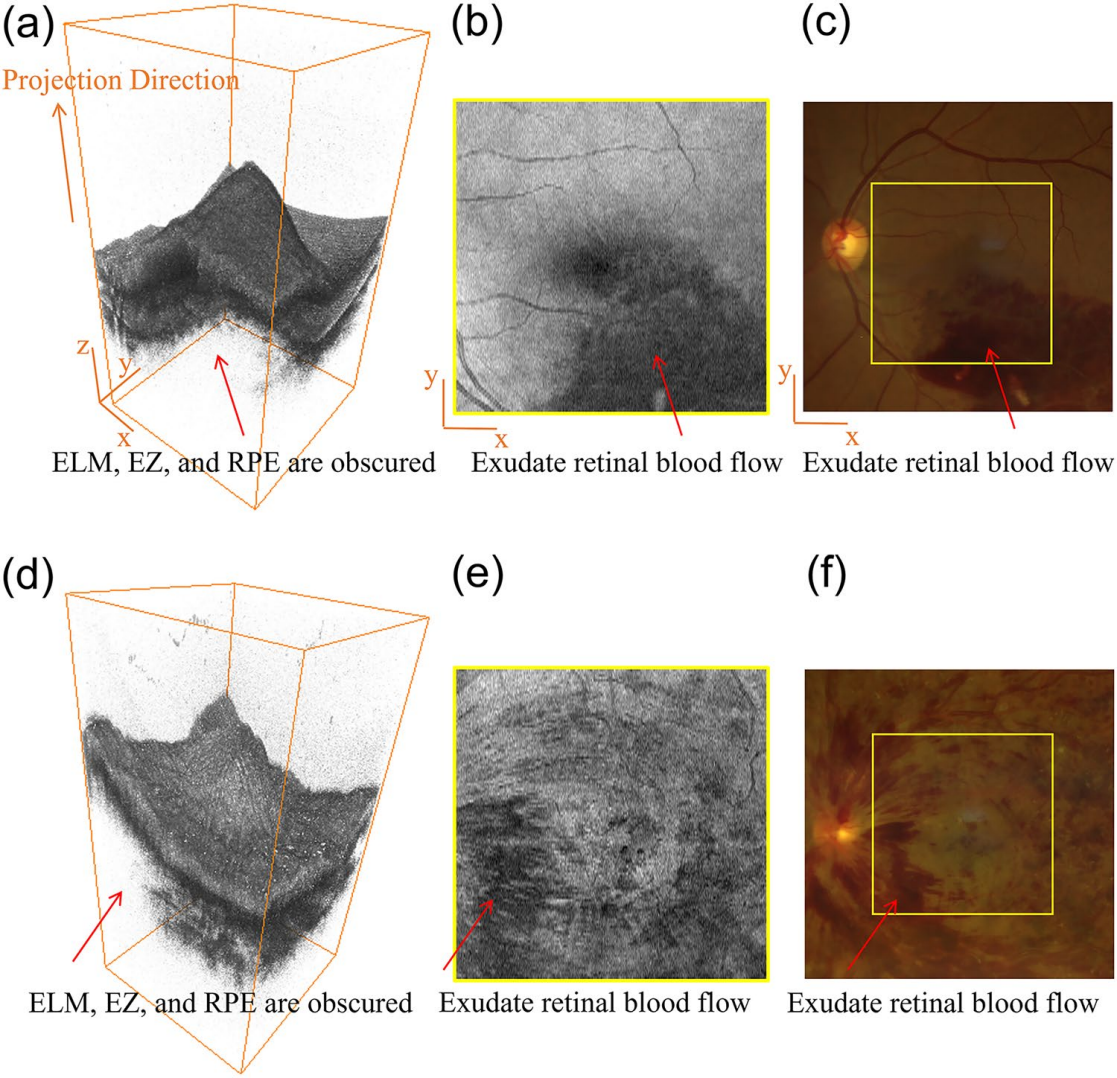
The underlying relationship between obscured retinal layers and exudate retinal blood flow. (**a**) a 3D rendering of a RVO volume. (**b**) a RVO projection map. (**c**) a RVO CFP. (**d**) a 3D rendering of a DR volume. (**e**) a DR projection map. (f) a DR CFP. DR = diabetic retinopathy; RVO = retinal vein occlusion; CFP = color fundus photography.

The pathogenesis of DME involves a combination of an inflammatory condition, altered vascular structures with increased membrane permeability, and secondary microaneurysm formation. DME can appear in two distinct patterns: diffuse edema and focal edema^21,22^. This diversity may arise from two different pathological mechanisms in DME: diffuse edema results from generalized capillary leakage, whereas focal edema is associated with microaneurysm (MA) leakage. The localized leakage leading to DME is attributed to MA leakage, while the diffuse leakage resulting in DME is believed to result from the dilatation of capillaries or small arteries within or near the leakage area^23^. In such cases, the edema appears more diffuse compared to leakage from MAs and gradually diminishes with increasing distance from the source. On the other hand, in RVO group, ME is believed to be caused by venous reflux obstruction resulting from tissue ischemia and hypoxia, elevated capillary hydrostatic pressure, and BRB breakdown. The blockage results in abnormal blood flow, increased venous pressure, and a higher chance of hemorrhage^24–26^. The exudate retinal blood flow blocks the retinal outer layers (shown in Fig. 5, the blocked retinal region in SD-OCT volume is consistent with the bleed region in color fundus photography (CFP)) as a result. A previous study has indicated that both BRVO and CRVO commonly manifest as generalized ME in horizontal volume scans^22^. This may be due to the relatively high incidence of temporal vein occlusion in BRVO, while the occlusion in CRVO involves the entire retinal vascular system, typically occurring near the optic disc thus macular region is influenced. We speculate that the differences in macular outer layer obscuration between RVO and DR can be related to the edema types (i.e., diffuse edema and focal edema) and hemorrhage caused by different pathologies. To be more specific, diffuse interstitial fluid accumulation leads to further thickening of the retina, resulting in unclear outer layer structures, while focal swelling has relatively less impact, allowing for clearer and more visible structures. Focal edema is more common in DME, whereas RVO tends to present with diffuse edema and a higher likelihood of hemorrhage. Therefore, the occlusion of retinal outer layers is observed more frequently in the RVO group.

HRF is also recognized as significant biomarker, and its distinct characteristics may reflect the pathogenic mechanisms of ME secondary to various etiologies^27^. Previous studies have indicated a higher propensity for the occurrence of HRF in DME, this could potentially be attributed to the more severe disruption of the BRB and a greater presence of hard exudates in the DME group^28^, which is consistent with our findings. Moreover, previous studies have demonstrated that the presence of HRF and the integrity of the ELM and EZ can serve as significant prognostic indicators for visual outcomes in patients with ME^29,30^.

We acknowledge several limitations of this study, including its retrospective design and the limited number of subjects. Prospective studies of larger sample size will be important to reaffirm the findings of this study. Besides, stratifying our cohort to analyze DROL and HRF features in subgroups generated unequal group sizes because the prevalence of BRVO is greatly higher than that of CRVO. We admit that the credibility of our findings in subgroup analysis can be compromised due to the predominance of BRVO cases. Another limitation is the lack of OCT and its corresponding CFP pairs (not all subjects involved in this study both took OCT and CFP imaging in a single visit) and we failed to analyze the underlying relationship between obscured retinal layers and exudate retinal blood flow in depth. Future study should collect the OCT-CFP paired data for further confirmation. Furthermore, we lack follow-up VA, limiting our ability to further analyze the correlation between indicators and long-term visual prognosis.

### Supplementary Information

Supplementary Information.

## Data Availability

The datasets used during the current study are available from the corresponding author on reasonable request.
