# Supplementary material for: Exploration on OCT biomarker candidate related to macular edema caused by diabetic retinopathy and retinal vein occlusion in SD-OCT images
Source: Sci Rep. 2024 Jun 21;14:14317. doi: 10.1038/s41598-024-63144-2 (PMC11192959; doi:10.1038/s41598-024-63144-2)
Supplement: Supplementary file 1 — Supplementary Information. [file 41598_2024_63144_MOESM1_ESM.docx]

**Supplementary Table S1**. Overview of the Entire Cohort.

| Disease | Subtype of Disease | Subjects (male/female) | Eyes (left/right) | Age(mean±std) |
| --- | --- | --- | --- | --- |
| DR | NPDR | 26 (16/10) | 32 (19 /13) | 61.62±10.12 |
|  | PDR | 61 (34/27) | 77(39/ 38) | 54.66±11.57 |
|  | Total | 87 (50/37) | 109 (58/51) | 56.74±11.55 |
| RVO | BRVO | 116 (56/60) | 120 (45/75) | 59.71±9.72 |
|  | CRVO | 15 (8/7) | 17 (7/10) | 61.60±12.74 |
|  | Total | 131 (64/67) | 137 (52/85) | 59.92±9.78 |
| DR = diabetic retinopathy; RVO = retinal vein occlusion; NPDR = non-proliferative DR; PDR = proliferative DR; BRVO = branch RVO; CRVO = central RVO; std = standard deviation. | | | | |

**Supplementary Table S2**. Evaluation Indicators Obtained by a 5-fold Cross-validation.

| Fold | Disease | # Correct / total samples | BACC(↑) | AUC(↑) |
| --- | --- | --- | --- | --- |
| 1 | DR | 17/20 | 0.9250 | 0.9857 |
|  | RVO | 28/28 |  |  |
| 2 | DR | 22/25 | 0.9043 | 0.9057 |
|  | RVO | 26/28 |  |  |
| 3 | DR | 20/21 | 0.8869 | 0.8827 |
|  | RVO | 23/28 |  |  |
| 4 | DR | 17/22 | 0.8864 | 0.9493 |
|  | RVO | 26/26 |  |  |
| 5 | DR | 18/21 | 0.9286 | 0.9630 |
|  | RVO | 27/27 |  |  |
| Total | DR  RVO | 94/109  130/137 | 0.9062 | 0.9373 |
| DR = diabetic retinopathy; RVO = retinal vein occlusion; BACC = balanced accuracy; AUC = area under the curve. | | | | |


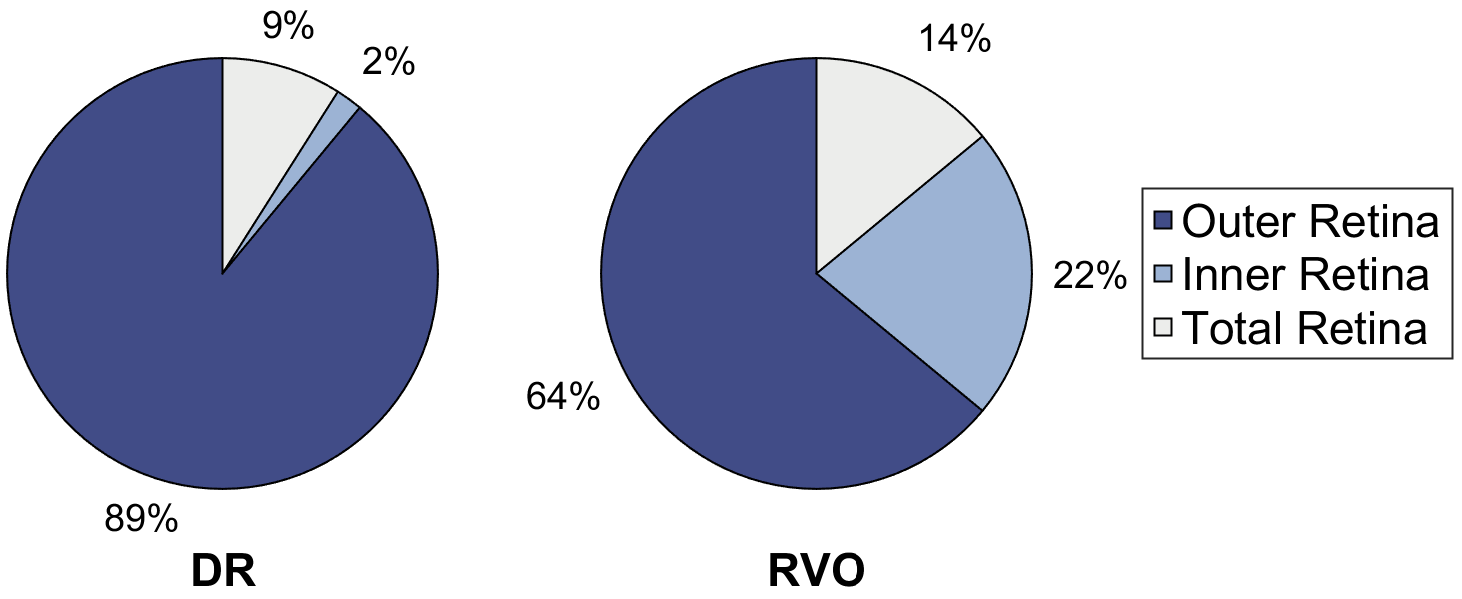


**Supplementary Figure S1.** Proportion of highlighted retinal regions of DR and RVO groups output by the DL model. DR = diabetic retinopathy; RVO = retinal vein occlusion; DL = deep learning.
